# Psychological Stress Deteriorates Skin Barrier Function by Activating 11β-Hydroxysteroid Dehydrogenase 1 and the HPA Axis

**DOI:** 10.1038/s41598-018-24653-z

**Published:** 2018-04-20

**Authors:** Sung Jay Choe, Donghye Kim, Eun Jung Kim, Joung-Sook Ahn, Eun-Jeong Choi, Eui Dong Son, Tae Ryong Lee, Eung Ho Choi

**Affiliations:** 1 Department of Dermatology, Yonsei University Wonju College of Medicine, Wonju, Korea; 2 Department of Psychiatry, Yonsei University Wonju College of Medicine, Wonju, Korea; 3 AmorePacific Corp/R&D Unit, Yongin, Korea

## Abstract

Psychological stress (PS) increases endogenous glucocorticoids (GC) by activating the hypothalamic-pituitary-adrenal axis. The negative effects of GC on skin barrier function under PS have been well-established. However, endogenous GC can also be active when cortisone (inactive form) is converted to cortisol (active form) by 11β-hydroxysteroid dehydrogenase type I (11ß-HSD1) in the peripheral tissue. Here, we evaluated the changes in 11ß-HSD1 and barrier function under PS. Elevated 11ß-HSD1 in oral mucosa correlated with increased cortisol in the stratum corneum and deteriorated barrier function. Expression of 11ß-HSD1 in the oral mucosa correlated with that in the epidermal keratinocytes. We further investigated whether barrier function improved when PS was relieved using a selective serotonin reuptake inhibitor (SSRI) in patients with anxiety. Decreased 11ß-HSD1 and improved barrier function were observed after SSRI treatment. The collective findings suggest that elevated 11ß-HSD1 under PS increases the level of cutaneous GC and eventually impairs barrier function. PS-alleviating drugs, such as SSRI, may help to treat PS-aggravated skin diseases.

## Introduction

Acute psychological stress (PS) due to various external threats and stimuli rapidly increases endogenous glucocorticoid (GC) levels and activates the autonomic nervous system (ANS), allowing the host to respond to various situations^1,2^. Many studies have already established the negative effects of PS on the skin. PS impairs the permeability barrier homeostasis^3^ and stratum corneum (SC) integrity^4^, and reduces both the innate and adaptive immunity of the epidermis^5,6^.

Although the changes of ANS and immunity are also important for these adverse effects on the skin under PS^7^, there is no doubt that the increase of endogenous GCs under PS plays a major role^8–10^. In humans, the hypothalamus-pituitary-adrenal (HPA) axis plays a major role in cortisol secretion. However, it has been reported that the peripheral HPA axis exists in various organs, including the skin. Keratinocytes also harbour homologues of all the major components of the HPA axis. Therefore, the skin acts as an endocrine organ^11^. In addition to the *de novo* synthesis of cortisol by the peripheral HPA axis in the skin, it has been reported that 11beta-hydroxysteroid dehydrogenase type 1 (11β-HSD1), which converts inactive cortisone into active cortisol, is present in the endoplasmic reticulum lumen of keratinocytes^12^. The role of 11β-HSD1 in the skin has been recently studied. 11β-HSD1 is associated with delayed wound healing in the skin and the inhibited proliferation of keratinocytes and fibroblasts^13–16^. Increased 11β-HSD1 upon ultraviolet (UV) irradiation reportedly correlates with transepidermal water loss (TEWL)^17^. In another study, UVB enhanced 11β-HSD1 gene and protein expression in a dose-dependent manner, and UVB and UVC enhanced cortisol production and decreased epidermal GR expression, while UVA had no detectable effects^18^. Others described that cutaneous GC genesis and cortisol signalling are defective in psoriasis, and that restoration of efficient endogenous GC signalling is a realistic goal in treating psoriasis^19^. A recent study also demonstrated that 11β-HSD1 inhibition can limit the cutaneous effects of GC excess, which may improve the safety profile of systemic steroids and the prognosis of chronic wounds^20^.

We hypothesised that the increase in 11β-HSD1 is a novel mechanism in the process of PS-related exacerbation of skin barrier dysfunction, that SC cortisol is a biomarker of PS, and that the aberrant skin barrier function can be restored when PS is relieved by the use of a selective serotonin reuptake inhibitor (SSRI) as a therapy for depression.

## Results

### PS-related skin barrier dysfunction is related to SC cortisol

**Figure 1:**
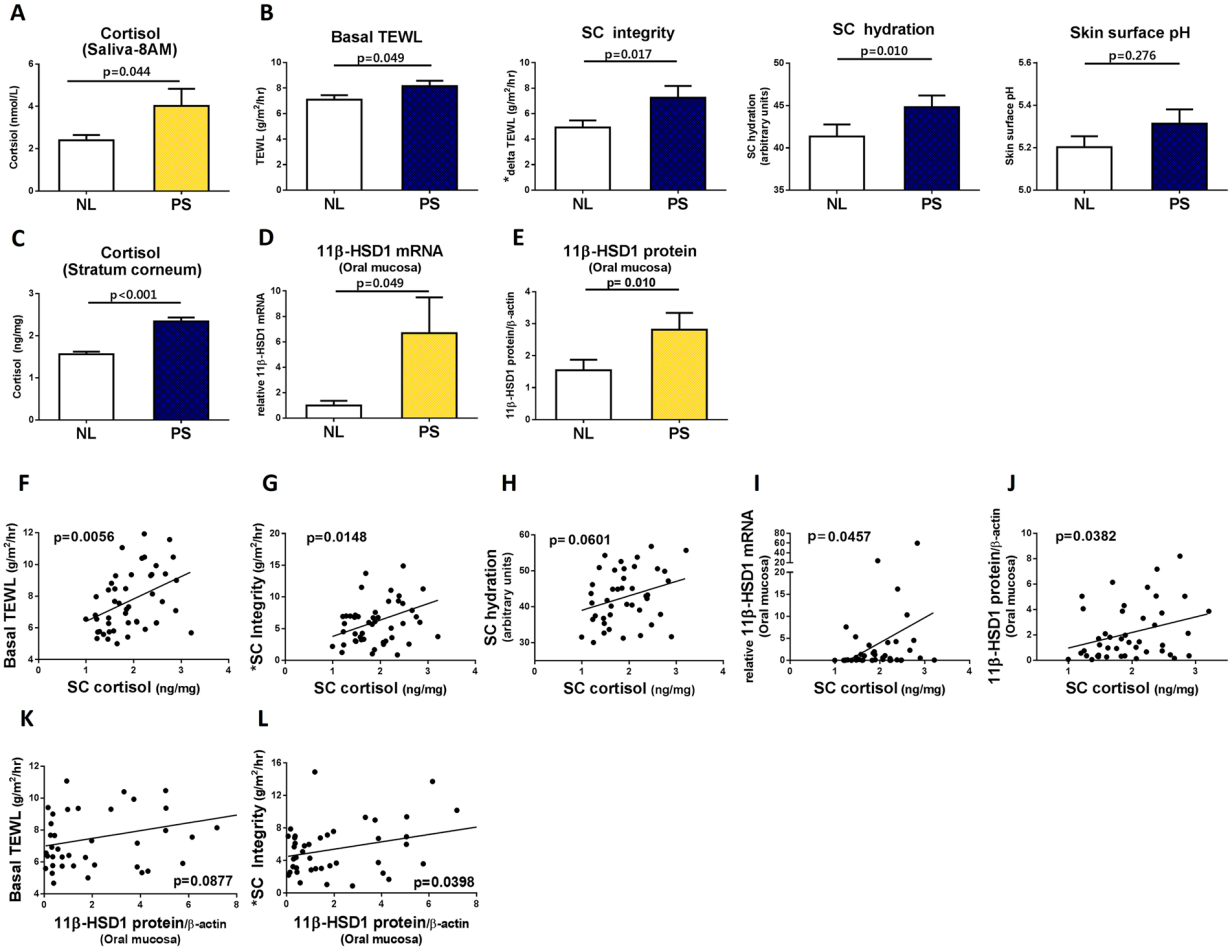
Accumulated cortisol of the stratum corneum and increased expression of 11β-HSD1 in oral epithelium may contribute to the deterioration of the skin barrier function under psychological stress (n = 25). (**A**) Basal salivary cortisol around 8AM increased under psychological stressed status (PS) compared to normal status (NL). (**B**) Skin barrier function deteriorated under PS. Basal TEWL and SC hydration increased. SC integrity indicated by delta TEWL was compromised under PS. Skin surface pH showed no significant difference between PS and NL. (**C**) Cortisol of the stratum corneum was increased under PS. **(D)** mRNA and (**E**) protein expression of 11β-HSD1 in oral mucosal epithelium increased under PS. Changes in skin barrier function ((**F**) Basal TEWL and (**G**) SC integrity**)**, and expression of 11β-HSD1 ((**I**) mRNA and (**J**) protein**)** were correlated with the levels of SC cortisol. Changes in **(H)** SC hydration also showed a positive correlation with them but not statistically significant. (**K**) Basal TEWL and (**L**) SC integrity were correlated with the levels of 11β-HSD1 but only SC integrity was statistically significant. *SC integrity = delta TEWL = PS TEWL (Post stripping with D-squame 15 times) - Basal TEWL.

PS was associated with higher levels of salivary cortisol 30 minutes after awakening (around 8AM) compared to the levels at that time during normal, non-stressed individuals (NL) (Fig. 1A). Concerning the skin barrier function, PS was associated with increases of basal TEWL and SC hydration increased, and a significant decrease of SC integrity (delta TEWL). Skin surface pH also tended to increase, but the increase was not significant (Fig. 1B). Cortisol was measured in the SC collected with tape stripping using D-squame. SC cortisol significantly increased under PS (Fig. 1C). SC cortisol levels were positively correlated with basal TEWL and SC integrity (Fig. 1F and G). SC hydration also tended to positively correlate with SC cortisol (Fig. 1H), but was not significant (p = 0.0601). Inflammatory cytokines were also measured in the collected SC. Interleukin (IL)-1α, IL-6, and tumour necrosis factor-alpha (TNF-α) levels were lower under PS compared to NL levels (Supplementary Fig. S1).

### Expression of 11β-HSD1 in oral epithelium increases under PS and is closely correlated with levels of SC cortisol and skin barrier function

Both mRNA and protein levels of 11β-HSD1 in oral mucosa increased under PS (Fig. 1D and E). The levels of SC cortisol positively correlated with the expressions of 11β-HSD1 mRNA and protein in the oral epithelium (Fig. 1I and J). The level of 11β-HSD1 protein in the oral epithelium tended to positively correlate with basal TEWL, but was not significant (Fig. 1K). However, the 11β-HSD1 protein level was significantly correlated with SC integrity (Fig. 1L).

### 11β-HSD1 expression in oral epithelium may be a marker of 11β-HSD1 expression in the epidermis

Since 11β-HSD1 is expressed primarily in the suprabasal layer of the epidermis^17^, its detection is possible only by an invasive method like skin biopsy, which collects the entire epidermis. Since the use of skin biopsy was not approved in the present study, we compared the expression of 11β-HSD1 in the epidermis with that of other tissues obtained non-invasively. The levels of 11β-HSD1 expressed in the keratinocytes from the foreskin obtained during circumcision and in oral mucosal epithelial cells obtained by oral swabbing were positively correlated in the same subjects.

**Figure 2:**
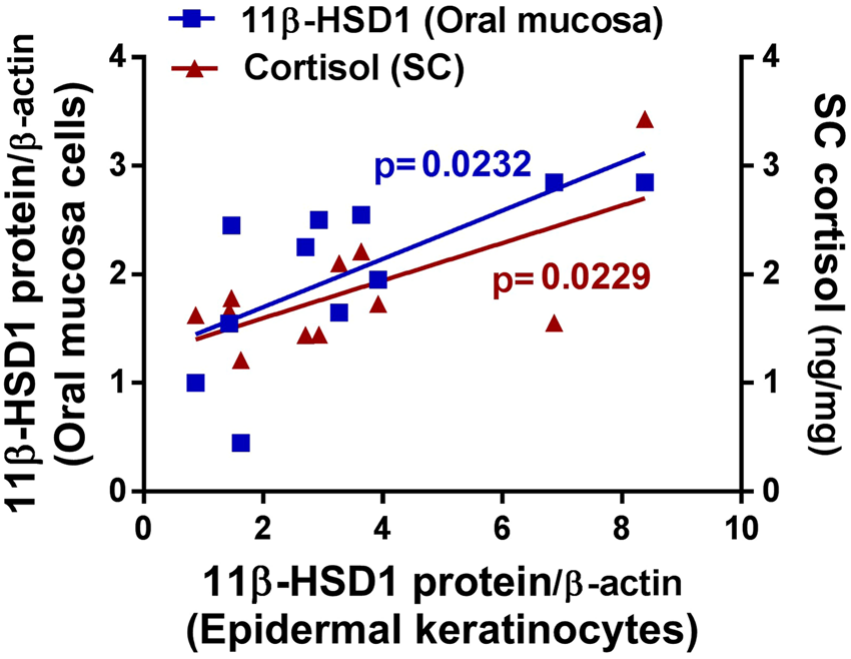
Expression of 11β-HSD1 in oral mucosal cells obtained by a non-invasive method may be used as a proxy for 11β-HSD1 expression in epidermal keratinocytes (n = 11). Foreskins were collected during circumcision. Oral mucosa was also collected by buccal swab just before surgery. 11β-HSD1 protein expression in the epidermis was well correlated with 11β-HSD1 protein expression in oral mucosa (blue) and cortisol of stratum corneum (red).

We then investigated whether SC cortisol correlated with epidermal 11β-HSD1 expression. The expression of 11β-HSD1 in the epidermis was positively correlated with the level of SC cortisol (Fig. 2). The finding indicates that measuring 11β-HSD1 in oral mucosal epithelial cells collected non-invasively method might replace the measurement of 11β-HSD1 from the epidermis.

### 11β-HSD1 activation increases cortisol production in keratinocytes *in vitro*

We needed to address whether the expression of 11β-HSD1 in keratinocytes was increased by elevated cortisol under PS. If so, the increased 11β-HSD1 during PS could convert inactive cortisone into active cortisol in the keratinocytes. To address this, we used keratinocytes transfected with HSD11B1-siRNA as a control. Ultraviolet irradiation is known to induce 11β-HSD1 and so was used here.

**Figure 3:**
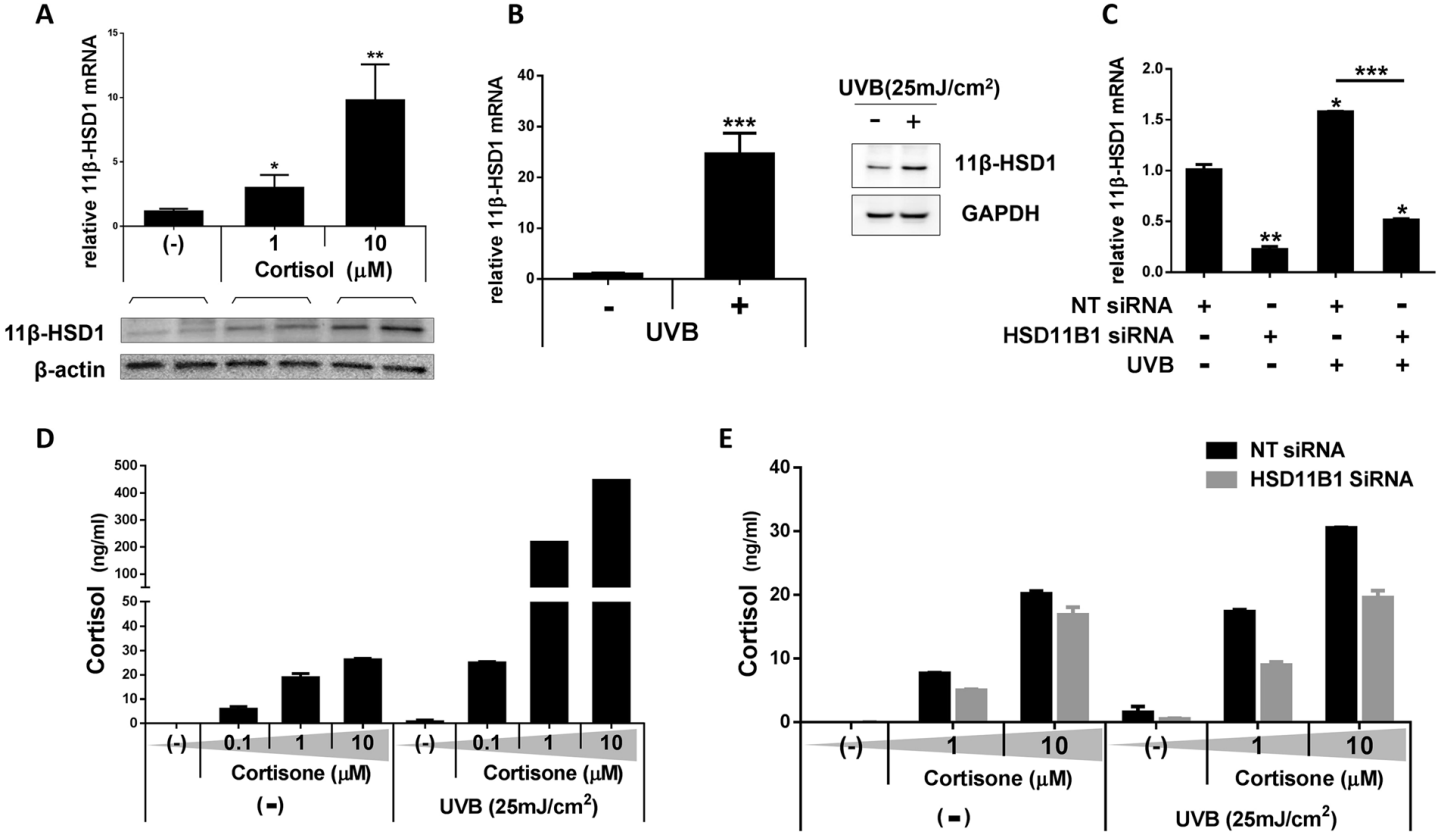
Expression of 11β-HSD1 is increased by cortisol itself and UV irradiation, thereby rapidly converting cortisone into cortisol (n = 3). (**A**) Primary human keratinocyte 11β-HSD1 mRNA and protein were upregulated dose-dependently by cortisol treatment. (**B**) 11β-HSD1 mRNA was approximately 25-fold higher in UVB irradiated primary human keratinocytes compared with control. (**C**) Keratinocytes transfected with HSD11B1-siRNA exhibited decreased 11β-HSD1 mRNA expression and this level did not increase, even when the keratinocytes were irradiated with UVB. (**D**,**E**) When keratinocytes were treated with cortisone, it was converted to cortisol in a dose-dependent manner. This conversion was more prominent under UV irradiation and decreased upon 11β-HSD1 knockdown. *p < 0.05; **p < 0.01; ***p < 0.001.

First, we observed that treating keratinocytes with cortisol dose-dependently increased mRNA and protein levels of 11β-HSD1 (Fig. 3A). In addition, when keratinocytes were irradiated with UVB, mRNA and protein expression of 11β-HSD1 increased significantly (Fig. 3B). The knockdown of 11β-HSD1 using small interfering RNA (siRNA) led to decreased 11β-HSD1 expression even under UVB irradiation (Fig. 3C). To evaluate the effect of 11β-HSD1 on endogenous GC metabolism in the skin, the changes in the level of cortisol produced from keratinocytes were evaluated upon cortisone treatment, UVB exposure, and knockdown of 11β-HSD1. The levels of cortisol increased even without UVB irradiation when cortisone was supplied, and the effect became more prominent with higher concentrations of cortisone. Cortisol levels were most significantly increased when cortisone was supplied along with UVB irradiation (Fig. 3D). In keratinocytes transfected with non-targeting (NT) siRNA, cortisol levels also increased after cortisone treatment with or without UVB irradiation. The levels of cortisol increased under UVB irradiation in keratinocytes transfected with HSD11B1 siRNA as the level of cortisone was increased. However, this effect was not prominent compared with keratinocytes transfected with NT siRNA (Fig. 3E).

### Cortisol treatment decreases keratinocyte differentiation *in vitro*

**Figure 4:**
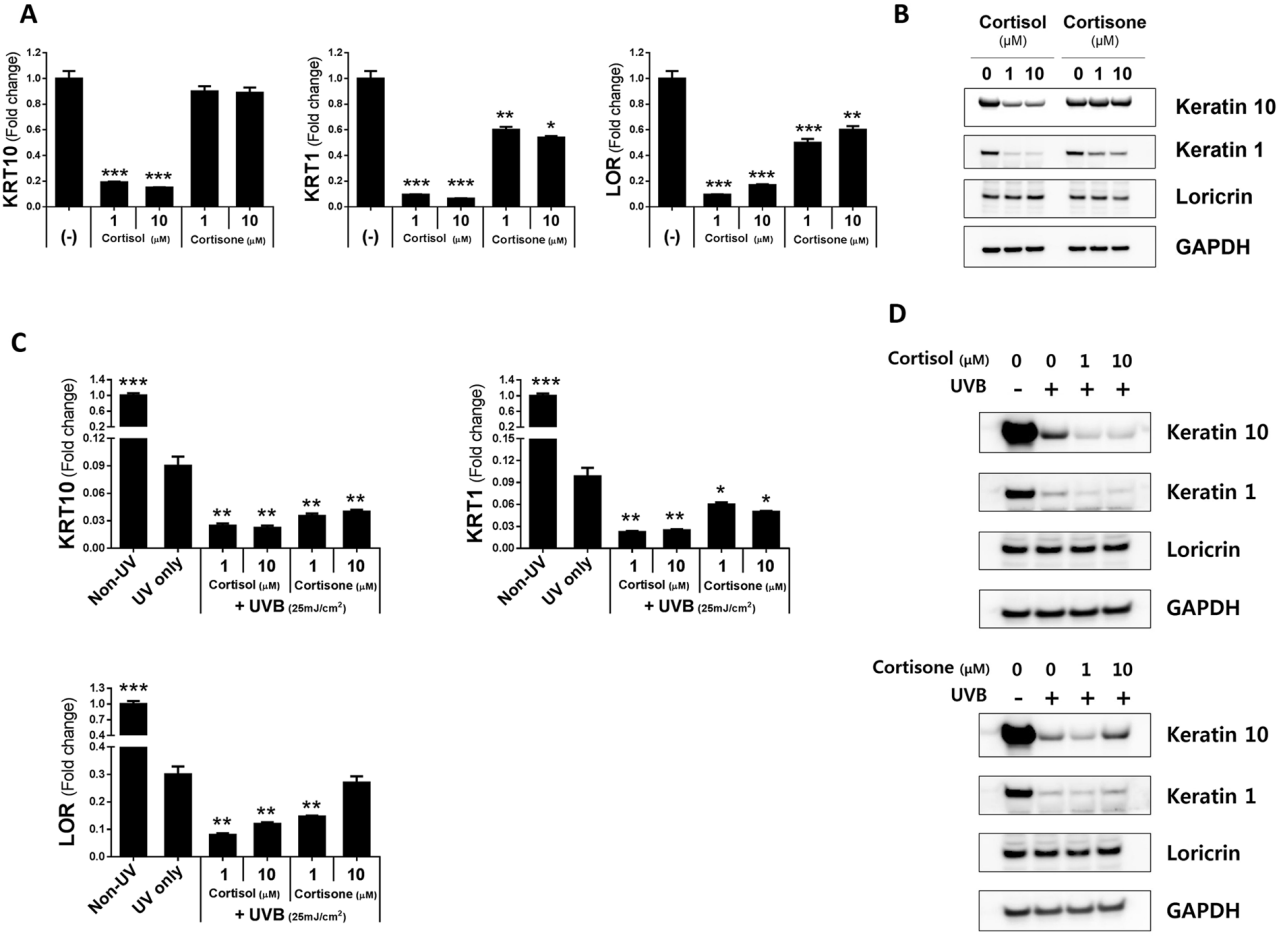
Increased cortisol converted by 11β-HSD1 inhibits keratinocyte differentiation *in vitro* (n = 3). (**A**) mRNA expression of keratinocyte differentiation markers (KRT10, KRT1, and LOR) was decreased by cortisol treatment, but was not affected or only slightly affected by cortisone. (**B**) Protein expression of KRT10 and KRT1 was decreased by cortisol, but the protein expression of LOR did not differ upon cortisol treatment. Protein expression of KRT10 and LOR was not affected by cortisone. Cortisone-induced protein expression of KRT1 decreased, but the change was less than that observed upon cortisol treatment at the same concentration. Cortisone decreased the mRNA (**C**) and protein (**D**) expression of keratinocyte differentiation markers (KRT10 and KRT1) in UVB-exposed keratinocytes. Cortisone decreased the mRNA expression of LOR but did not alter its protein expression in UVB-exposed keratinocytes. *p < 0.05; **p < 0.01; ***p < 0.001.

We additionally investigated the effect of cortisol on keratinocyte differentiation. Cortisol treatment of normal human keratinocyte (NHK) cells resulted in a dramatic reduction of mRNA expression of KRT10, KRT1, and loricrin. These changes were also observed with 1 µM cortisol. However, cortisone treatment did not result in any change in KRT10 even with treatment up to 10 µM, although slight decreases in KRT1 and loricrin were observed (Fig. 4A). After cortisol treatment, protein levels of KRT10 and KRT1 decreased, but loricrin levels showed no difference. Cortisone treatment did not affect KRT10 protein expression. However, KRT1 protein expression was decreased after cortisone treatment. This decrease was less than the decrease observed at the same concentration of cortisol. There was no difference in protein expression of loricrin regardless of cortisone or cortisol (Fig. 4B). The same experiment was repeated using UVB irradiation. The expressions of KRT10, KRT1, and loricrin in keratinocytes decreased after irradiation. The decreased expression of the differentiation markers most prominent upon co-treated with cortisol, with marked decreases also observed with co-treatment with cortisone compared to treatment with cortisone only (Fig. 4C). Protein expressions of KRT10 and KRT1 were decreased in UVB-irradiated keratinocytes. When cells were exposed to both cortisol and UVB irradiation, the expressions of KRT10 and KRT1 were more prominently decreased compared with their expressions in the absence of irradiation. Cortisone treatment and UVB irradiation also reduced the expressions of KRT10 and KRT1 compared with keratinocytes that were not irradiated. However, there was no change in loricrin expression regardless of UVB irradiation and treatment with cortisone and cortisol (Fig. 4D).

### SC cortisol is regulated by 11β-HSD1 activation in epidermal keratinocytes and is mainly affected by cortisol in the SC intercellular space

Using foreskins removed from circumcised patients, we compared the changes in cortisol under UVB irradiation with or without the application of a topical 11β-HSD1 inhibitor. The increased protein expression of 11β-HSD1 in UVB-irrradiated cells was confirmed by immunohistochemical staining and western blot (Supplementary Fig. S2A and B). SC cortisol was also increased upon UVB irradiation. The levels recovered to normal levels when a topical 11β-HSD1 inhibitor was applied (Supplementary Fig. S2C). The SC is largely composed of corneocytes and the intercorneocyte space (ICS). Methanol can be used to extract SC intercellular lipids from the SC (Kim *et al*., 2017). Therefore, we adopted this method to separate corneocytes and SC intercellular components.

Cortisol was separately measured in the proteins extracted from the ICS and in the separated corneocytes. There was no significant change in the cortisol level in corneocytes regardless of UVB irradiation and application of 11β-HSD1 inhibitor (Supplementary Fig. S2D). However, the altered levels of cortisol in the ICS and epidermis were similar to its changes in the SC (Supplementary Fig. S2E and F). Furthermore, expression of 11β-HSD1 in the epidermis was positively correlated with the cortisol levels of the entire SC, ICS, and epidermis (Supplementary Fig. S2G and H).

### Anti-depressant treatment recovers skin barrier function impaired by elevated epidermal cortisol and 11β-HSD1 activation in patients with depression

**Figure 5:**
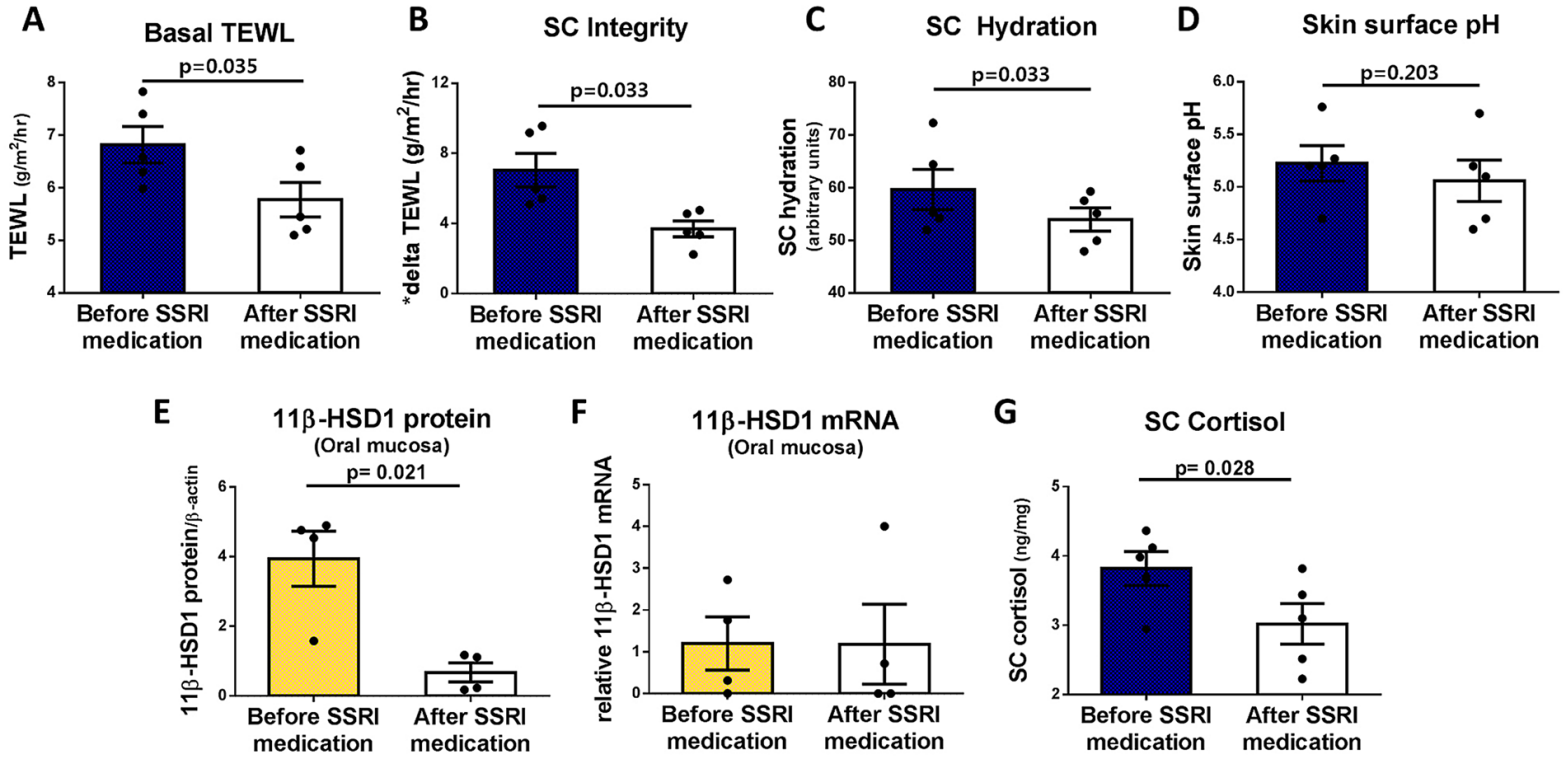
Anti-depressant (SSRI) treatment ameliorates depression and recovers skin barrier function impaired by elevated epidermal cortisol and 11β-HSD1 activation. (**A**) Basal TEWL, (**B**) SC integrity, and (**C**) SC hydration decreased after treatment with the SSRI escitalopram versus before medication (n = 5). (**D**) Skin surface pH also tended to decrease after SSRI medication treatment, but statistical significance was not observed. (**E**) Protein and (**F**) mRNA expression of 11β-HSD1 and (**G**) cortisol of the stratum corneum decreased after SSRI medication versus before medication. *SC integrity = delta TEWL = PS TEWL (Post stripping with D-squame 15 times) - Basal TEWL.

For patients who were initially diagnosed with depression by a psychiatrist, skin barrier function and the expressions of 11β-HSD1 in oral mucosal cells and SC cortisol were compared before treatment and after the 6-week administration of selective serotonin reuptake inhibitor (SSRI). Basal TEWL and SC hydration decreased and SC integrity improved after treatment with escitalopram (Fig. 5A–C). The pH of the skin surface also decreased after the treatment, but the decrease was not significant (Fig. 5D). Protein expression of 11β-HSD1 decreased after the administration of SSRI, but there was no significant change in mRNA expression (Fig. 5E and F). Cortisol in the SC also decreased significantly after SSRI administration (Fig. 5G). The changes in 11β-HSD1 expression in oral mucosa after SSRI administration showed a significant positive correlation with the SC cortisol and a negative correlation with SC integrity (Supplementary Fig. S3A and B). Only a positive tendency with SC hydration was observed (p = 0.0546) (Supplementary Fig. S3C). The changes in SC cortisol after SSRI administration also showed a negative tendency with SC integrity (p = 0.0698) (Supplementary Fig. S3D).

## Discussion

Examination stress is a valid form of PS and SC cortisol is a potential biomarker of PS. Examination stress has been shown to be an appropriate PS model^3,21^. Under PS, increased morning cortisol and deterioration of skin barrier function were observed, similar to the results of a previous study^22^. Presently, SC cortisol and 11β-HSD1 of oral mucosal cells increased under PS. We quantified cortisol in the SC and found that the cortisol levels were closely related to the skin barrier function under PS. Furthermore, changes in both mRNA and protein of 11β-HSD1 in oral mucosal cells were positively correlated with SC cortisol level (Fig. 1A–J).

Increased 11β-HSD1 could be a novel mechanism of PS exacerbating barrier functions. The observation that the expression of the 11β-HSD1 protein itself was also negatively correlated with skin barrier function under PS (Fig. 1K and L) indicate that 11β-HSD1 acts as a key molecule in the skin to regulate the level of cortisol, and that elevated cortisol deteriorates skin barrier function. Since it is practically difficult to evaluate the changes in 11β-HSD1 in human skin, we attempted to find an indirect and non-invasive method to measure 11β-HSD1 expression in the skin. Consequently, 11β-HSD1 expression in keratinocytes strongly correlated with 11β-HSD1 expression in oral mucosal epithelial cells, and also correlated with SC cortisol (Fig. 2). Thus, even though the oral mucosa is far from the skin, the changes in 11β-HSD1 associated with PS, including examination-related stress and depression were closely related to changes in skin barrier function and SC cortisol (Figs 1 and 5). These results suggest that the level of 11β-HSD1 in epidermal keratinocytes could be estimated indirectly using oral mucosal epithelial cells collected non-invasively, and that this protein may be useful as a PS biomarker.

11β-HSD1 expressed in normal keratinocytes actively converts cortisone to cortisol. Various factors including endocrines, inflammation, and metabolic factors influence the expression of 11β-HSD1, and are often transient or tissue-specific^23,24^. In the skin, the expression of 11β-HSD1 increases with increasing endogenous GC in sebocytes and fibroblasts^25,26^, similar to a variety of other cells^23^. We also observed the increased content of 11β-HSD1 in keratinocytes as the cortisol concentration increased. Furthermore, the amount of cortisol produced by NT siRNA-transfected keratinocytes was higher than that of keratinocytes transfected with HSD11B1 siRNA, even without UVB irradiation. This difference indirectly suggests that increased expression of 11β-HSD1 was due to the positive feedback by cortisol. It is unclear which mechanism directly regulates 11β-HSD1 expression in various conditions, but it is clear that the action of 11β-HSD1 is important in skin cortisol metabolism for the following reasons. First, increased cortisol in SC and epidermis after UV irradiation were restored to normal levels after applying the 11β-HSD1 inhibitor (Supplementary Fig. S2C). Second, the increase in cortisol was significantly greater in the cortisone-treated group than in the control group regardless of UVB irradiation. Moreover, the increase in cortisol under UVB irradiation was markedly reduced in keratinocytes transfected with HSD11B1 siRNA compared to keratinocytes transfected with NT siRNA (Fig. 3A,D,E).

Cortisol decreases differentiation markers involved in skin barrier function. Expressions of the differentiation markers KRT10 and KRT1 were decreased with increased cortisol levels. KRT10 and KRT1 proteins are expressed during early differentiation processes in keratinocytes^27^. Since these differentiation markers play an important role in maintaining skin integrity and permeability barrier function, their decrease in the presence of increasing concentrations of cortisol is directly related to the deterioration of skin barrier function^28,29^. In our study, the changes in mRNA expression of loricrin were similar to those of both KRT10 and KRT1, but there was no difference in protein expression. This is because loricrin is expressed during late differentiation, and therefore more time is needed to induce the difference in protein expression (Fig. 4). Several previous studies have also shown that topical steroid application or endogenous GC exacerbates skin barrier function^4,10^, and reduces keratinocyte differentiation and proliferation.

Pro-inflammatory cytokines in the SC are decreased under PS. Our results (Supplementary Fig. S1) are consistent with the decreased expression of pro-inflammatory cytokines (IL-1β, IL-8, TNF-α, etc.) when wound healing is delayed due to stress-induced GC production under PS^30^. Indeed, in the normal skin, cytokine expression plays an important role in regulating skin homeostasis, including proliferation and differentiation^31–34^. Therefore, it can be considered that increased cortisol levels that occur under PS decrease the expression of homeostasis regulation cytokines, rather than influencing inflammation, thereby contributing to the deterioration of the skin barrier function. However, it has been reported that the expression of pro/anti-inflammatory cytokines, such as TNF-α, IL-β, and IL-10, in serum increases under PS^18^, in contrast to the observations in the skin.

11β-HSD1 may increase cortisol levels in keratinocytes, leading to excretion of cortisol and its diffusion into the SC intercellular space. To explore the origin of SC cortisol, we observed changes in epidermal and SC cortisol under UVB irradiation, which increases 11β-HSD1 and cortisol. In addition, since SC consists of corneocytes and ICS, we separated them for analysis. There was no significant change in corneocyte cortisol (Supplementary Fig. S3D), suggesting that acute stress was not reflected in corneocytes. This is probably because 11β-HSD1 is mainly expressed in the suprabasal layer of the epidermis and because it takes several days for a keratinocyte to differentiate into a corneocyte. In contrast, a dramatic alteration of cortisol that was evident in the ICS (Supplementary Fig. S3E) suggested that cortisol, which is mainly converted from cortisone at the epidermis, diffuses into the SC. Additionally, UVB irradiation or supplementation of culture media with cortisone increased the cortisol level in the media (Fig. 3D and E).

Increased SC hydration under PS may be related to activation of eccrine glands via ANS activation. SC hydration increased under PS in the participants and decreased after use of SSRI in those with depression (Figs 1B and 5C). These observations indicate that SC hydration is closely related to PS. In response to various stimuli, sympathetic nerves are activated and release catecholamine that affects the innervated skin. PS promotes adrenal catecholamine release in addition to GC production by the HPA axis^35^. Catecholamine is involved in the immune response and cell differentiation in the skin^36^, but it also acts on the eccrine gland to increase sweating^37^. Furthermore, in a study of PS and skin symptoms in college students, hyperhidrosis was 2.56 times higher in the high-stress group than in the low-stress group^38^.

Skin barrier function can be restored when PS is relieved by SSRI. The skin barrier function was improved in patients with depression after 6 weeks of SSRI therapy (Fig. 5A–D). Others reported that barrier recovery was delayed in mice under PS, and was restored by pharmacologically reducing PS with tranquiliser therapy using diazepam and chlorpromazine^39^. In addition, barrier recovery is improved in mice and humans by inhaling an odour with a sedative effect^40^. These results suggest that when barrier function is exacerbated under PS, modifying the PS by medication could be helpful for restoring barrier function. In addition to alleviating PS and changing in skin barrier function, psychiatric medication may be effective in various skin disorders that are exacerbated upon PS^41,42^. In patients with anxiety or depression, dysregulation of the HPA axis leads to high basal cortisol levels in the serum^43,44^. Although the mechanism is still unclear, SSRIs or various sedative drugs, including escitalopram, restore HPA axis function to normal in patients with generalised anxiety disorder and depression^45,46^. This process is presumed to be related to the restoration of the damaged barrier in patients with depression. Considering the increase in cortisol level and elevation of 11β-HSD1 under PS in normal subjects, decreased expression of 11β-HSD1 after SSRI medication in the patients with depression (Fig. 5E) may be attributed to the normalisation of the the HPA axis and the consequent reduction of basal cortisol level. The small number of patients with diagnosed depression is an acknowledged important limitation. It was difficult to persuade depressed patients visiting the psychiatry outpatient clinic to agree to participate without any guarantee of therapeutic benefit. This was a preliminary study, which will hopefully be the basis of future clinical studies with larger numbers of patients.

PS acts as an aggravation factor in various dermatological diseases. Especially, chronic inflammatory skin diseases, such as atopic dermatitis (AD) and psoriasis (PSO), are easily aggravated by PS^36,47^. Indeed, both AD and PSO are associated with psychiatric disorders, such as anxiety and depression^48^. In patients with the severe and chronic AD and PSO, the HPA axis is blunted and endogenous GC does not rise sufficiently in stressful situations^36^. This may result in the flare-up of these diseases under PS. However, since the use of SSRIs, which have relatively few side effects when used properly, can help normalise the HPA axis and allow it to respond appropriately in stress situations, these drugs could be effective in preventing the exacerbation of the disease under PS.

Collectively, under PS, the systemic HPA axis is activated and serum and skin cortisol levels are increased. These events increase the expression of 11β-HSD1, thereby amplifying the cortisol level in the skin. Increased cortisol inhibits the differentiation of keratinocytes and decreases the expression of cytokines needed to maintain the barrier function, with the ultimate deterioration of the skin barrier function (Supplementary Fig. S3). The observation that skin barrier function recovered when the PS was relieved by SSRI treatment suggests the potential value of SSRIs for patients with skin diseases exacerbated by PS. Changes in barrier function associated with PS are closely related to the changes in SC cortisol and 11β-HSD1 in oral mucosal cells, which can be easily and non-invasively collected and used as PS biomarkers.

## Materials and Methods

This study was approved by the Yonsei University Wonju Campus Institutional Review Board and was performed in accordance with their guidelines (CR315014, CR316025, CR317026). All participants provided written informed consent.

### Medical students before and after examination

Twenty-seven medical students were recruited through advertising. Two were excluded due to eczema lesions. The remaining 25 provided their informed consent to participate and were enrolled (CR315014). All participants were male medical students in the same grade. They were all in good health. The examination model was adopted as a type of PS. NL was defined as the period without an examination for two weeks before and after, and PS was defined as the period during final examinations (Table 1). The final examinations were scheduled on five consecutive days. All PS measurements were made on the fourth day. All measurements were also repeated under NL.

**Table 1:** Baseline features of the 25 participating male students.

Age (years)	Body mass index (kg/m^2^)	Sleeping time during non-exam (hours) - NL	Sleeping time during exam (hours) - PS
20.2 ± 1.3*	24.3 ± 3.6	6.74 ± 1.05	3.92 ± 1.57

*Mean ± SD.

### Patients with depression before and after SSRI medication

Five patients were initially diagnosed to suffer from depression by a psychiatrist at Wonju Severance Christian Hospital, using Diagnostic and Statistical Manual of Mental Disorders Fifth Edition (DSM-5) criteria for a depressive episode and the Depression Anxiety Stress Scale (DASS-21). The patients provided informed consent to participate (CR316025). Patients diagnosed as depression were treated for 6 weeks with the SSRI escitalopram oxalate. All measurements were acquired before treatment (before treatment (before psychiatric [PSY] medication) and after treatment (after PSY medication).

### Measurement of skin barrier function

Skin barrier functional parameters of basal TEWL, SC hydration, skin surface pH, SC integrity, and delta TEWL after 15 D-squame detachments were evaluated in all participants. Basal TEWL was measured with a Tewameter TM210 apparatus (Courage and Khazaka, Cologne, Germany), and SC hydration was assessed as capacitance with a Corneometer CM820 device (Coruage and Khazaka). Skin surface pH was measured with a pH meter (WTW, Weilheim, Germany).

### Cell culture

Normal human epidermal keratinocytes (NHEKs; Lonza, Basel, Switzerland) within two or three passages were cultured in KBM-Gold medium supplemented with KGM-Gold Bullet Kit (Lonza) at 37 °C and 5% CO_2_. Hydrocortisone, one of the components of the KGM-Gold Bullet kit, was not added to the medium for this assay. NHEKs were seeded in 6-well plates and cultured in complete growth medium. After 24 h, the cells were treated with cortisol (Sigma-Aldrich, St. Louis, MO, USA) or cortisone (Sigma-Aldrich) in the assay medium and harvested 4 days later for further analysis. In irradiation experiments, NHEKs were irradiated with UVB at 25 mJ/cm^2^ using a Biosun UV irradiation system (Vilber Lourmat, Marnes-la-Valle-e, France) before treatment with cortisol or cortisone. For siRNA experiments, NHEKs were transfected with 50 nM of non-targeting control (NT) siRNA (Bioneer, Daejeon, South Korea) or siRNA against 11β-HSD1 (Bioneer) using Lipofectamine RNAimax transfection reagent (Thermo Fisher Scientific, Loughborough, UK). At 24 h post-transfection, the cells were irradiated with UVB (25 mJ/cm^2^) followed by treatment with cortisol (10 μM) or cortisone (10 μM). After 4 days, the cells and culture supernatants were harvested for RT-qPCR and ELISA analyses.

### Organ culture with foreskin

Eleven patients who underwent circumcision in urology surgery provided informed consent to participate in this study (CR317026). The foreskin was removed during circumcision and oral mucosal cells were collected. The expression of 11β-HSD1 was compared between the epidermal keratinocytes and the oral mucosal cells. Five foreskin samples were equally divided into three parts. One part was irradiated with sham light and applied with vehicle. Another part was irradiated with UVB and applied with vehicle. The third part was UVB-irradiated and applied with topical 11β-HSD1 inhibitor. All skin samples for organ cultures were obtained from the foreskins of 11–12-year-old circumcised children. Skin tissues were divided into sections, rinsed with PBS, and cultured in an air-medium interface at 37 °C under a 5% CO_2_ atmosphere. The culture medium consisted of DMEM supplemented with 10% FCS, 1 mg/mL ciprofloxacin, and 200 mM l-glutamine. To promote the separation of epidermis from dermis, skin sections were incubated with 15 U/mL dispase for 1 h at 37 °C. The epidermis was isolated using forceps. The expression of 11β-HSD1, SC cortisol, epidermal cortisol, and cortisone were measured. In addition to the entire SC, cortisol in the corneocytes and the intercellular spaces were measured separately.

### Treatment with 11β-HSD1 inhibitor

11β-HSD1 inhibitor (CAS 1009373-58-3) purchased from Merck Millipore (Billerica, MA, USA) is a potent inhibitor of 11β-HSD1. The inhibitor was dissolved in dimethylsulfoxide in a 1:1 mixture for topical application. Dimethylsulfoxide (DMSO) was used as a vehicle control.

### Quantification of cortisol by ELISA

Each ELISA assay was performed according to the manufacturer’s protocol. All standards and samples were measured in duplicate.

#### Salivary cortisol

Subjects were instructed to obtain saliva within 30 minutes after waking up. Subjects were instructed to tilt their heads slightly forward and to accumulate the saliva in the floor of mouth before spitting the saliva into a conical tube. The samples were kept frozen by subjects themselves, and then the samples were stored at −70 °C until analysis. Salivary cortisol was analysed using an enzyme immunoassay kit (Salimetrics, Carlsbad, CA, USA).

#### Stratum corneum cortisol

D-squame disc tapes (CuDerm, Dallas, TX, USA) were attached to the forearm skin to collect stratum corneum from the medical students and patients with depression. In organ culture model experiments, SC was collected by attaching D-squame disc tapes to the foreskin directly. Five strips were used to extract proteins. Strips were placed in 500 μL of lysis buffer, followed by vortexing and overnight incubation at 4 °C. Especially in organ cultured skins, the five collected strips were cut into two equal pieces. Half of the strip was treated with methanol to dissolve intercellular lipids and cortisol to compare the cortisol between corneocytes and ICS. The other half was used to measure the cortisol of whole SC. The amount of cortisol in extracted proteins was measured with the cortisol ELISA kit (Merck Millipore, Darmstadt, Germany).

#### Epidermal (keratinocyte) cortisol and cortisone

In the organ-cultured skin, the epidermis and dermis were separated using EDTA. Protein was extracted from the separated epidermal samples, and cortisol and cortisone were quantified. In the keratinocyte culture, cortisol was quantified in the media after 96 h of incubation after each treatment. The amount of cortisol was measured by a cortisol ELISA kit (R&D Systems, Minneapolis, MN, USA) according to the manufacturer’s instructions.

### Cytokine multiplex analysis

SC samples of subjects were frozen and thawed only once before performing the MILLIPLEX MAP human cytokine/chemokine panel (Merck Millipore, Billerica, MA, USA), a bead-based multiplex immunoassay, which allows the simultaneous quantification of IL-1α, TNF-α, and IL-6. SC samples were processed in duplicate following the manufacturer’s recommended protocols and read on a MAGPIX instrument equipped with the MILLIPLEX-Analyst software using a five-parameter nonlinear regression formula to compute sample concentrations from the standard curves.

### Real-time reverse transcriptase-PCR

Total RNA was isolated from the cultured cells and tissue was powdered according to the protocol supplied with QIAzol reagent (RNeasy lipid tissue kit, QIAGEN Inc., Valencia, CA, USA) according to the manufacturer’s instructions. The RNA was then quantified using a NanoDrop spectrophotometer (Thermo Scientific, Wilmington, DE), and 4 μg of the sample was used as a template for cDNA synthesis by reverse transcription with a SuperScript reverse transcriptase III kit (Invitrogen, Carlsbad, CA, USA). Real-time PCR was performed in a solution (20 μL) containing 2 TaqMan universal PCR mixture (10 μL), 20X TaqMan expression assay mix (1 μL), cDNA (50 ng), and primers. qRT-PCR was performed using a 7500 Fast Real-Time PCR system (Applied Biosystems, Foster City, CA). The conditions for thermal cycling were as follows: initial denaturation for 5 minutes, followed by 45 cycles of amplification at 95 °C for 15 s, annealing at 60 °C for 30 s, and extension at 76 °C for 30 s. After the PCR was complete, the cycle threshold (CT) value of each gene was verified and analysed using ΔΔCT. TaqMan probes for qPCR (Applied Biosystems) were HSD11B1, KRT10, KRT1, Loricrin, and RPL13A, using probes Hs00194153_m1, Hs0016628 9_m1, Hs00196158_m1, Hs01894962_s1*, and Hs04194366_g1, respectively.

### Western blot

Cell samples were extracted using RIPA cell lysis buffer (QIAGEN Inc.) containing a protease and phosphatase inhibitor cocktail. Twenty-five micrograms of total protein were used for western blotting following the standard protocol. Proteins were separated by electrophoresis on 10% SDS-PAGE gels and transferred to PVDF membranes (GE Healthcare, Pittsburgh, PA, USA). The membranes were blocked with 5% non-fat milk in Tris-buffered saline and Tween 20 (TBST) for 1 h, then incubated overnight at 4 °C with anti-11β-HSD1 (Santa Cruz Biotechnology, Santa Cruz, CA, USA), anti-KRT1 (BioLegend, San Diego, CA, USA), anti-KRT10 (BioLegend), anti-LOR (BioLegend), glyceraldehyde-3-phosphate dehydrogenase (GAPDH; Santa Cruz Biotechnology), and β-actin (Abcam, Cambridge, UK). All membranes were washed three times with TBST and bound antibodies were sequentially detected by proper secondary antibody conjugated to horseradish peroxidase (Cell Signaling Technology, Danvers, MA, USA) at room temperature (22 ± 2 °C) for 2 h. Antibody-bound proteins were visualised using ECL substrate (GE Healthcare).

### Immunohistochemistry

Immunohistochemical staining was performed to assess expression of 11β-HSD1 protein. Briefly, 5 μm-thick paraffin sections were incubated with primary antibodies against 11β-HSD1 proteins (Santa Cruz Biotechnology) overnight at 4 °C. After three cycles of washing, the sections were incubated with the anti-rabbit secondary antibody for 30 min. Staining was detected with an ABC-peroxidase kit (Vector Lab, Burlingame, CA, USA), and counter-staining with haematoxylin was performed. Representative images for each group were provided.

### Statistical analyses

Statistical analyses were performed using GraphPad Prism software (San Diego, CA, USA). Student’s *t*-test or paired *t*-test was used for intergroup comparison, repeated measures ANOVA was used for the repeated measures, and linear regression was used to compare the correlation between variables. Data represent the mean ± SEM. p < 0.05 was considered statistically significant.

## Electronic supplementary material

Supplementary information
