# Supplementary material for: Psychological Stress Deteriorates Skin Barrier Function by Activating 11β-Hydroxysteroid Dehydrogenase 1 and the HPA Axis
Source: Sci Rep. 2018 Apr 20;8:6334. doi: 10.1038/s41598-018-24653-z (PMC5910426; doi:10.1038/s41598-018-24653-z)

Supplementary Information

Psychological Stress Deteriorates Skin Barrier Function by Activating 11β-Hydroxysteroid Dehydrogenase 1 and the HPA Axis

Sung Jay Choe[*], Donghye Kim[*], Eun Jung Kim[*], Joung-Sook Ahn[†], Eun-Jeong Choi[‡], Eui Dong Son[‡], Tae Ryong Lee[‡], Eung Ho Choi[*]

[*]*Department of Dermatology, Yonsei University Wonju College of Medicine, Wonju, Korea,*

[†]*Department of Psychiatry, Yonsei University Wonju College of Medicine, Wonju, Korea,*

[‡]*AmorePacific Corp/R&D Unit, Yongin, Korea*

Supplementary Figure S1. Expression of inflammatory cytokines in the stratum corneum was decreased under PS. **(A)** IL-1α, **(B)** TNF-α, and **(C)** IL-6 expression was decreased under PS compared to NL.

Supplementary Figure S2. SC cortisol is regulated by 11β-HSD1 activation in epidermal keratinocytes and is mainly affected by cortisol in SC the intercellular space. Protein expression of 11β-HSD1 increased in the both UV-exposed skins compared to sham-light exposed skin. **(A)** Epidermal 11β-HSD1 staining intensity appeared greater in UV-exposed skin versus sham-light exposed skin under de-melanin stain. **(B)** Western blot image quantification confirmed increased expression of 11β-HSD1 in UV-exposed skin versus sham-light exposed skin. **(C)** Cortisol of the stratum corneum was increased in "UVB+Veh" skin compared to "sham-light+Veh" and "UVB+11β-HSD1 inh" skins. **(D)** No significant difference in the cortisol of the corneocyte was observed due to UV irradiation or 11β-HSD1 inhibitor application. Changes in cortisol of the intercorneocyte space **(E)** and epidermis **(F)** were similar to those in the whole stratum corneum (C). **(G)** When the 11β-HSD1 inhibitor applied group was excluded, 11β-HSD1 protein expression in the epidermis was positively correlated with cortisol in the whole stratum corneum (blue), and intercorneocyte space (black) but was not correlated with corneocyte levels (red). **(H)** 11β-HSD1 protein expression in the epidermis was also positively correlated with cortisol in the epidermis (orange).

Supplementary Figure S3. Expression of 11β-HSD1 in oral mucosal cells of patients with anxiety disorder or depression negatively correlated with skin barrier function and positively with SC cortisol before and after SSRI treatment.
Expression of 11β-HSD1 protein was positively correlated with the levels of SC cortisol **(A)** and negatively correlated with SC integrity **(B).** Changes in SC hydration **(C)** also showed positive correlation with 11β-HSD1 protein level but this effect was not statistically significant. The levels of SC cortisol positively correlated with SC integrity but this effect was not statistically significant **(D)**. *SC integrity = delta TEWL= PS TEWL (Post stripping with D-squame 15 times) - Basal TEWL.

Supplementary Figure S1. Expression of inflammatory cytokines in the stratum corneum was decreased under PS (n=25).

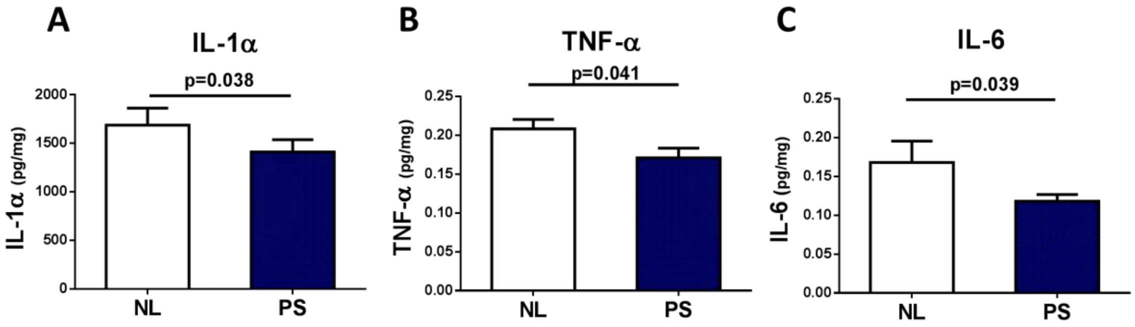

Supplementary Figure S2. SC cortisol is regulated by 11β-HSD1 activation in epidermal keratinocytes and is mainly affected by cortisol in SC the intercellular space (n=5).

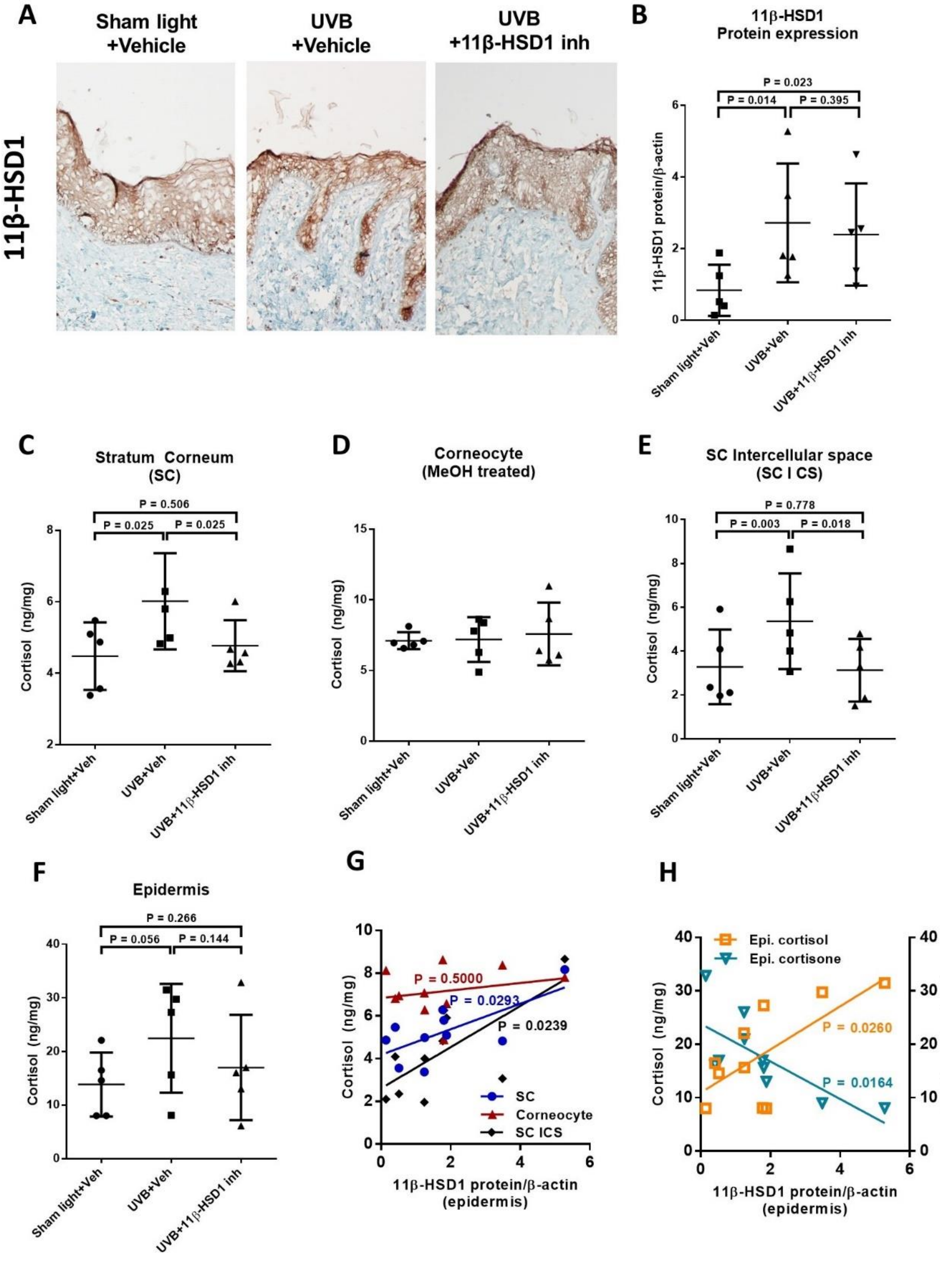

Supplementary Figure S3. Expression of 11β-HSD1 in oral mucosal cells of patients with anxiety disorder or depression negatively correlated with skin barrier function and positively with SC cortisol before and after SSRI treatment.

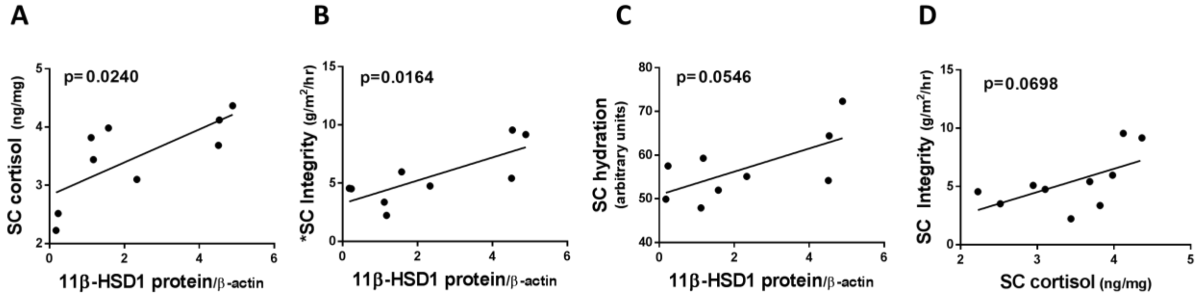

Supplement: Supplementary file 1 — Supplementary information [file 41598_2018_24653_MOESM1_ESM.pdf]
